# Serotherapy against Voltage-Gated Sodium Channel-Targeting α-Toxins from *Androctonus* Scorpion Venom

**DOI:** 10.3390/toxins11020063

**Published:** 2019-01-23

**Authors:** Marie-France Martin-Eauclaire, Sonia Adi-Bessalem, Djelila Hammoudi-Triki, Fatima Laraba-Djebari, Pierre E. Bougis

**Affiliations:** 1 Laboratory of Cognitive Neuroscience, CNRS, Aix Marseille Univ, UMR 7291, 13003 Marseille, France; marie-france.eauclaire@univ-amu.fr; 2 Laboratory of Cellular and Molecular Biology, Faculty of Biological Sciences, USTHB, BP 32, El-Alia Bab Ezzouar, 16111 Algiers, Algeria; sbessalem@usthb.dz (S.A.-B.); dhammoudi@usthb.dz (D.H.-T.); flaraba@usthb.dz (F.L.-D.)

**Keywords:** *Androctonus*, scorpion, venom, toxin, Nav channel, serotherapy

## Abstract

Because of their venom lethality towards mammals, scorpions of the *Androctonus* genus are considered a critical threat to human health in North Africa. Several decades of exploration have led to a comprehensive inventory of their venom components at chemical, pharmacological, and immunological levels. Typically, these venoms contain selective and high affinity ligands for the voltage-gated sodium (Na_v_) and potassium (K_v_) channels that dictate cellular excitability. In the well-studied *Androctonus australis* and *Androctonus mauretanicus* venoms, almost all the lethality in mammals is due to the so-called α-toxins. These peptides commonly delay the fast inactivation process of Na_v_ channels, which leads to increased sodium entry and a subsequent cell membrane depolarization. Markedly, their neutralization by specific antisera has been shown to completely inhibit the venom’s lethal activity, because they are not only the most abundant venom peptide but also the most fatal. However, the structural and antigenic polymorphisms in the α-toxin family pose challenges to the design of efficient serotherapies. In this review, we discuss past and present accomplishments to improve serotherapy against *Androctonus* scorpion stings.

## 1. Introduction

Scorpions comprise around 2400 species, and belong to an ancient and ecologically successful group of animals that has been exemplified in the fossil record for some 400 million years [1,2]. They pose a public health threat in many countries, but particularly in North Africa [3,4]. Scorpions most dangerous for humans belong to the *Buthidae* family, which, with 82 genera and 756 species, is the largest scorpion family, found on every continent except Antarctica [5,6]. About twenty *Buthidae* species are known to be lethal to humans. Some of these lethal species belong to the *Androctonus* genus, as *A. australis* in Algeria (*Hector* morph) and in Tunisia (*garzonii* sub-species) and *A. mauretanicus* in Morocco. These sizeable animals can inoculate up to 500 µg of a venom that is particularly rich in toxins. In the Maghreb, these two species are responsible for about 100,000 stings per year and, 1 to 7% lead to death [7]. Their median lethal dose (LD_50_) by subcutaneous (*s.c*.) injection is between 1 and 5 µg per mouse (20 g), and they are therefore considered to be the most lethal scorpion species in the world for mammals and humans [8,9]. Other venomous *Androctonus* species are *Androctonus amoreuxi*, *Androctonus aeneas aenaes*, *Androctonus crassicauda*, and *Androctonus bicolor*. However, the chemical and pharmacological properties of their venoms are still poorly studied, compared to what is currently known about the *Androctonus australis* and *Androctonus mauretanicus* venoms. The three Pasteur Institutes in Maghreb have largely added to our knowledge of the *Androctonus mauretanicus* and *Androctonus australis* venoms, their main objective being the production of specific and efficient antivenoms for serotherapy purposes [10,11,12]. 

Victims of scorpion stings suffer various pathologies, involving both sympathetic and parasympathetic stimulation as well as central manifestations such as irritability, hyperthermia, vomiting, profuse salivation, tremor, and convulsions. The clinical signs and symptoms observed in humans and experimental animals are related to an excessive systemic host inflammatory response to stings. In addition to cardiac dysfunction, pulmonary edema, and respiratory failure, systemic inflammatory response seems to be strongly implicated in the pathogenesis of scorpion envenomation. The complexity of scorpion pathogenesis and its severity reduces the efficacy of treatment. Thus, improving serotherapy is a key challenge for scientists and antiserum producers.

Scorpion venoms are complex mixtures of peptides and proteins, for which many have yet to be assigned a function. The polypeptide toxins from scorpion venom have very specific actions, and mainly interact with different ion channels and receptors in excitable membranes. Four different families of scorpion neurotoxins have been described, which specifically recognize voltage-gated sodium, voltage-gated potassium, voltage-gated calcium, and chloride channels [13]. These neurotoxins are present in the venom as a few percent of the dried venom weight. In *Androctonus* venoms, s.c. venom toxicity in mammals has mainly been attributed to the activity of long polypeptide chain toxins, which bind with high affinity to voltage-gated sodium (Na_v_) channels [14,15]. Indeed, Na_v_ channels are very critical for generating the rising phase of an action potential by promoting a rapid flux of ions across the membrane [16], an action that is disrupted by scorpion toxins. With their high number of disulfide bonds (four), which hold together their rather small molecular size (60–70 residues), these toxins can persist in a hostile environment because they are highly stable and resistant to denaturation. They display a high degree of relatedness at the level of three-dimensional (3D) structure, despite having more limited sequence homology.

Neutralization of scorpion venoms by heterologous antivenoms has been extensively investigated. However, the effectiveness of each commercial available antivenom, produced in a different geographical area, in neutralizing homologous and heterologous scorpion venoms has been a matter of debate [17]. Nowadays, antivenom specificity can be explained by the large amount of chemical and immunological data accumulated so far.

In this review, we will tackle recent research progress that led to our understanding of (1) the mechanisms contributing to the pathophysiology and inflammatory response after envenomation, (2) the chemistry of *Androctonus* venom α-toxins and their immunochemical interrelations, and (3) the set-up of an appropriate serotherapy with the most recent developments, and possible future directions.

## 2. Immediate Envenomation Symptoms

Commonly, the symptoms of scorpion stings are mainly observed in the peripheral nervous system. Stings in children, the elderly, and immunocompromised people are much more dangerous than in healthy adults. Following a sting, symptom progression is rapid. However, serotherapy is very effective when a specific antiserum is rapidly injected; victims typically recover within one hour after administration.

Three stages of severity are described [18]. First, an immediate intense and persistent pain (up to two hours) is the dominant clinical sign. During this unthreatening stage I, other discrete general symptoms can be observed such as agitation, febricula, sweats, nausea, feeling of general faintness, and alternating blood pressure (hypertension or hypotension). During stage II, which is considered a severe envenomation, the body temperature increases and sweats, epigastric pain, vomiting, colic, diarrhea, priapism, hypotension, bradycardia, pulmonary obstruction, and dyspnea can appear. Vomiting indicates huge severity and necessitates specific monitoring. Stage III is only seen in 5–10% of stage II cases, and is potentially fatal. At this late stage, cardiac arrhythmia and myocardial ischemia explain the risk of cardiovascular collapse, associated with severe respiratory complications such as pulmonary edema, bronchospasm, and cyanosis.

## 3. *Androctonus* Genus Venoms Involved in Complex Systemic Inflammatory Response Syndrome

### 3.1. Scorpion Venom and Inflammatory Response

Scorpion envenomation is considered to be one of the non-infectious diseases that can evolve into severe cases of systemic inflammatory response syndrome [19,20,21]. Indeed, the venom components diffuse rapidly from the vascular compartment blood to organs and several signaling pathways that are involved in the induced pathogenesis after stings. Within the first minutes after a sting, the pathophysiology of envenomation involves a highly integrated response, including the activation of various cell types that release a spectrum of chemical mediators ultimately affecting various target tissues. The biological disorders occurring after scorpion envenomation are not only the consequence of the discharge of neurotransmitters such as catecholamine and acetylcholine; they can also be attributed to other pathophysiological mechanisms, including increased blood vessel permeability and the establishment of an immuno-inflammatory response. The immuno-inflammatory process observed after envenomation involves a set of local and peripheral cellular and molecular mediators that amplify the biological disorder [22,23,24]. Excessive immune responses induce multiple organ dysfunctions in envenomed patients and/or experimental animals to a large extent, and contribute to strong inflammatory response. 

**Figure 1:**
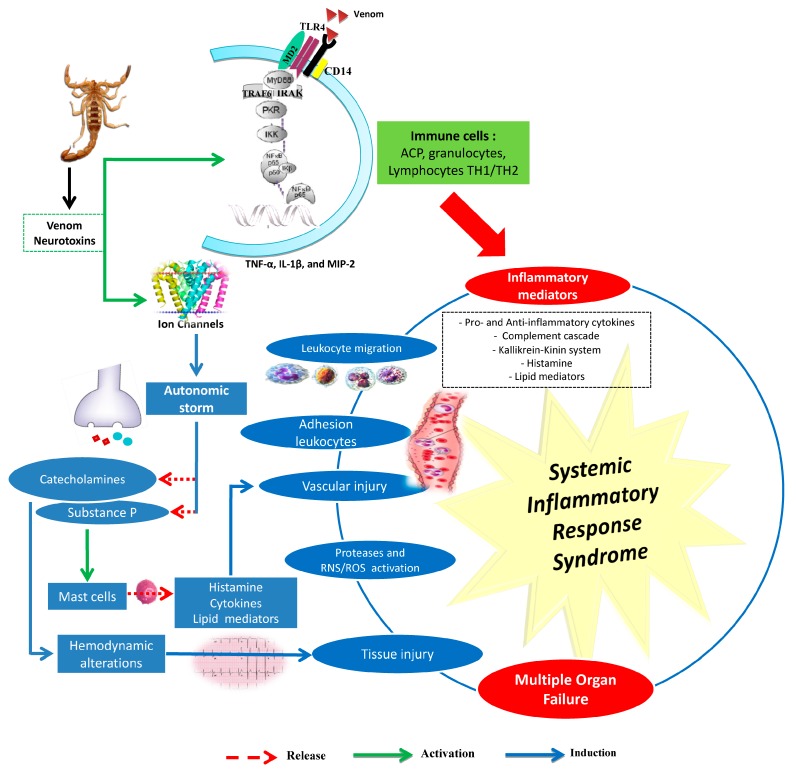
Main events involved in the induced inflammatory response by scorpion venoms. *RNS*: Reactive nitrogen species; *ROS*: Reactive oxygen species. The main effects caused by scorpion venoms or by their neurotoxins are mainly due to the release of mediators from the autonomic nervous system (catecholamines and substance P) and from immune cells (cytokines, lipid mediators, reactive oxygen species, and nitric oxide). These mediators promote systemic inflammatory response syndrome (SIRS), causing multiple organ failure (MOF). Drawn according to data issued from [20,26,27,29,30,31,32,33,34,35,36,37,38,39,40].

*Androctonus australis hector* venom and its toxins (e.g., Aah I and Aah II) are able to induce leukocyte recruitment and an inflammatory response in several animal model (rats, mice, and rabbits) tissues (lungs, kidneys, liver, heart, stomach, and intestine) [20,25,26,27,28,29,30,31,32,33]. Intensive leukocyte mobilization and infiltration into organs are associated with the development of multitude organ failure and high mortality rates, especially among children. These immune defense cells release a group of secreted mediators and other signaling molecules (e.g., cytokines, histamine, serotonin, eicosanoids, and activation of the complement and kallikrein–kinin and oxygen- and nitrogen-derived free radicals, etc.) (Figure 1).

Cytokines play a critical role in orchestrating, perpetuating, and amplifying the inflammatory response in scorpion envenomation [41]. Most are involved in immune reactivity, tissue injury, or repair and organ dysfunction. Clinical and experimental data demonstrate a causal relationship between overproduction of certain cytokines, such as interleukin-1β (IL-1β), interleukin-8 (IL-8) and interleukin-6 (IL-6), with the severity of symptoms, serum venom concentration, and morbidity/mortality [26,28,42,43,44,45].

*Androctonus australis hector* venom increases pro-inflammatory (IL-1β, IL-6, TNF-α) and anti-inflammatory cytokine levels (IL-4, IL-10) and activates the complement system, effects that are correlated with tissue damage [20,35]. Scorpion envenomation also induces other cytokines (IL-4, IL-5, and IFNϒ) as well as lipid-derived mediators of inflammation, including platelet activator factor (PAF), leukotrienes, and prostaglandins [20,46,47,48] (Figure 1). Indeed, bioactive lipids play a critical role in initiating and resolving inflammation. It has been suggested that PLA2 and derivatives are involved in the induced vasodilatation and edema formation, inflammatory cell chemotaxis, and subsequent tissue damage by Aah venom [49]. Experimental studies have also revealed that an increase of kinin or histamine levels exacerbates the inflammatory response as observed after Aah scorpion envenomation [29,32]. These mediators can promote the migration of polymorphonuclear cells associated with the formation of tissue edema, and induce the production of mediators such as cytokines, reactive oxygen species, prostaglandins, and leukotrienes [50,51].

Finally, scorpion-sting-induced human envenomation provokes an intense inflammatory reaction [52]. However, the mechanisms by which the innate or adaptive immune system detect the venom components and initiate inflammatory processes and the signaling pathways that regulate the immune response remain largely unknown.

### 3.2. Involvement of the Innate Immune System 

Immune defense plays an important role in immuno-treatment of the host. As innate immune is the major system that contributes to the inflammation response, immune cells such as macrophages, dendritic cells, mast cells, neutrophils, and lymphocytes play important roles in the progression of the inflammatory response. Moreover, scorpion-induced pathogenesis is a dynamic process, in which the risk of septic shock, and therefore outcome, is determined by the balance between destructive inflammatory cell activity and the reparative effects. Activation of Toll-like receptors (TLR) receptors by venom components induces the production of inflammatory mediators such as inflammatory cytokines TNF-α, IL-1, and IL-6 and chemokines. These mediators rapidly accelerate the progression of inflammation through the modification of vascular endothelial permeability, as well as the recruitment of neutrophils and excess plasma containing antibodies and complement components into the site of injury. Furthermore, studies have reported that the interaction of *Tityus serrulatus* venom (TsV) with Toll-like receptors 2 (TLR2) and TLR4 could induce activation of signaling pathways involving a range of transcriptional factors (NF-κB and AP-1, ERK1/2 and p38) that are responsible for an immune response [52]. Similarly, the use of different protein kinase inhibitors that target elements downstream of TLR signaling has shown that TAK1, IKκ-β, and MEK regulate TNF-α, IL-1β, and MIP-2 expression on alveolar macrophage cells in response to Aah venom [38]. All these pathways play critical roles at multiple levels within the immune system, including the inflammatory response and cell cycle progression.

Envenomation by Aah venom is also able to induce systemic release of granulocyte colony-stimulating factor (G-CSF), macrophage CSF (M-CSF), IL-8, and monocyte chemo tactic protein 1 (MCP-1) [37]. Indeed, these mediators can induce systemic bioactivity following venom diffusion and are not only anti-inflammatory cytokines, but also colony-stimulating factors and chemokines, which were secreted in an earlier phase of envenomation. Although the wider pathological implications are still not well understood, an increase of cytokine levels may, at least in part, explain the augmented cell-mediated immunity and allergic reactions derived from a higher type-1 to type-2 cytokine ratio, along with mobilization and functional amplification of neutrophils and monocytes [31,34,38,53].

### 3.3. Oxidative Stress and Scorpion Pathogenesis

Oxidative stress caused by overproduction of reactive oxygen species (ROS) has been shown to be an important factor in the pathogenesis of scorpion envenomation [26,54,55]. Infiltrated inflammatory cells in different tissues of envenomed animals produce ROS that interfere with nitric oxide (NO) and form reactive species derived from NO. NO is an important free radical, serving as a second messenger in processes including neurotransmission, maintenance of vasodilator tone, and arterial pressure, and it has been suggested that cytokine-mediated circulatory shock is caused by activation of the inducible isorform (type II) of NOS [26]. A correlation between the release of NO and the severity of scorpion envenomation has been shown to exist [56]; [26,57,58]. This increase of NO and ROS is associated with lipid peroxidation in tissues. Indeed, nitrogen and oxygen species can react with cell membrane fatty acids to impair their function. The major product of lipid peroxidation, the MDA, may amplify the immunopathology response [23,59]. Elevated lipid peroxidation during Aah scorpion envenomation in different tissues has also been reported [26,40,49,55]. The reactive species can induce inflammation through activation of transcription factor NF-*κ*B. Furthermore, oxidative stress plays an important role in the activation of NOD-like receptor protein 3 (NLRP3) inflammasome [60]. The produced radical reactive species, such as the superoxide anion, released at the inflammatory site can also cause the activation of matrix metalloproteases (MMPs) [61]. Indeed, an increase in the expression of matrix metalloproteases (2 and 9) has also been observed after administration of Aah venom to animals [29,40]. The deregulation of protease expression leads to the destruction of a large amount of tissue, including connective tissue and basement membrane, resulting in various inflammatory pathological conditions (Figure 1).

## 4. Antivenoms and Immunological Properties of Scorpion Toxins

### 4.1. Antivenoms

The majority of antivenom producers use the direct injection of crude venom into horses. Optimal antibody production typically takes up to six weeks. Recently, camel immunization (*Camelus dromedarious*) was also described [62]. In order to obtain a less immunogenic product and increase human compatibility or tolerance, varying manufacturer efforts have been made to remove animal proteins such as albumin. The antidotal fraction of a commercially antivenom exists as either whole IgG, Fab, or F(ab)2. Although Fab and F(ab)2 are more expensive to produce, as opposed to their whole immunoglobulin counterparts, they are generally regarded as less allergenic and therefore safer to use. IgG is the easiest and cheapest to produce. Because of its size, it is less filterable at the glomerulus and has the smallest distribution volume. IgG has a longer elimination half-life than either Fab or F(ab)2 [63]. F(ab)2 has less potential to initiate anaphylaxis. Fab is the smallest (50 kDa) in size, and thus has the largest volume of distribution and a greater ability to reach intracellular compartments before being eliminated by the kidney [63]. Immunoglobulin-based serotherapy can be given by intramuscular, intravenous, or subcutaneous route.

### 4.2. Characterization of Toxins at The Pharmacological, Structural, and Immunological Level

#### 4.2.1. Pharmacology of *Androctonus* α-Toxins Targeting Na_v_ Channels 

Almost all (90%) of the total toxicity of *Androctonus* venom towards mice is attributed to the long chain toxins that are able to bind with high affinity to Na_v_ channels and act as gating modifiers [14,64]. Na_v_ channels are transmembrane proteins that are crucial for generating action potentials by opening and closing rapidly [65]. Scorpion toxins specific for Na_v_ channels have been divided into two major classes: α-toxins, the major lethal components of Old World scorpion venoms like *Androctonus* venoms, and β-toxins, found in majority in New World scorpion venoms. *Androctonus* α-toxins bind to voltage-sensing domain IV (VSD IV) of the Na_v_ channel and hold it in its inward position, thereby inhibiting the fast inactivation process of the channel. Consequently, a transient increase in Na^+^ permeability disturbs the action potential by inducing a persistent depolarization of the cell membrane [16,66]. Their binding is dependent on membrane potential, and decreases at depolarized potential [67,68].

#### 4.2.2. Structure of *Androctonus* α-Toxins Targeting Na_v_ Channels

**Figure 2:**
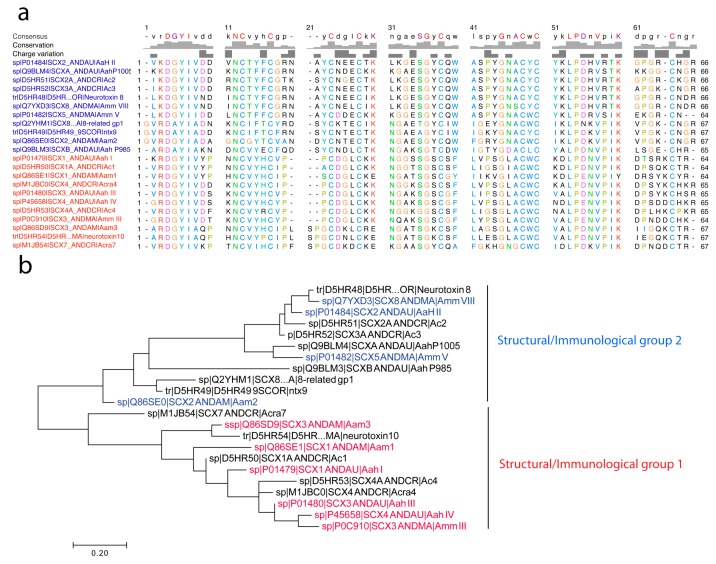
Molecular phylogenetic analysis by maximum likelihood method of *Androctonus* α-toxins. (**a**) The evolutionary history was inferred by using the maximum likelihood method, based on the Jones–Taylor–Thornton (JTT) matrix-based model [69]. (**b**) The tree with the highest log likelihood (−1310.1986) is shown, displaying two major structural/immunological groups (clades) as noted. Initial tree(s) for the heuristic search were obtained automatically by applying Neighbor-Join and BioNJ algorithms to a matrix of pairwise distances estimated using a JTT model, and then selecting the topology with superior log likelihood value. The tree is drawn to scale, with branch lengths measured in the number of substitutions per site. The analysis involved 22 amino acid sequences taken from UniProtKB, for which accession numbers are reported (i.e., D5HR48, etc.). All positions containing gaps and missing data were eliminated. There was a total of 62 positions in the final dataset. Evolutionary analyses were conducted in MEGA7 [70]. Of note, the presence of GR at the N-terminal is the signal peptide for the α-amidation processing of the toxin precursor n-3 amino acid residue [71]. Otherwise, ultimate R residues are not present in mature toxins, as exemplified for the sequences of Amm VII and Amm III that have been obtained using Edman sequencing.

Na_v_ channel-specific *Androctonus* α-toxins are mono-concatenated peptides of 60 to 70 amino acid residues cross-linked by four disulfide bridges (Cys–Cys). In Figure 2 we report the molecular phylogenetic analysis by maximum likelihood method of 22 primary structures so far described for *Androctonus* α-toxins, obtained either by Edman degradation from purified toxins or deduced from cDNA sequencing and genes encoding homologous putative toxins (collected at Expasy Bioinformatics Resource Portal).

The *Androctonus* Na_v_ channel toxins have an α/β scaffold (βαββ), containing one α-helix and a three-stranded anti parallel β-sheet, connected by three loops of different sizes and highly variable compositions [72,73,74]. These secondary elements play a major role in the constitution of the toxin’s epitopes (see following section). Three disulfide bridges tightly fold the α-helix and the β-sheets and stabilize the core of the toxins: Cys^3^ and Cys^4^ bind Cys^6^ and Cys^7^ in the β3 strand, Cys^5^ in the β2 strand binds Cys^2^. Finally, Cys^1^ links Cys^8^ and connects the N-terminal to the C-terminal. All these toxins exhibit a large solvent-exposed conserved hydrophobic surface, made by a cluster of aromatic and hydrophobic residues [73,75]. Structural studies of scorpion toxins have shown subtle differences in the spatial arrangement of the turn preceding Cys^1^ (N region), the C-terminal region, and the region between Cys^5^ and Cys^6^ (β2–β3 loop).

#### 4.2.3. First Immunological Characterization of *Androctonus* α-Toxins: Definition of the Structural and Immunological Groups

**Figure 3:**
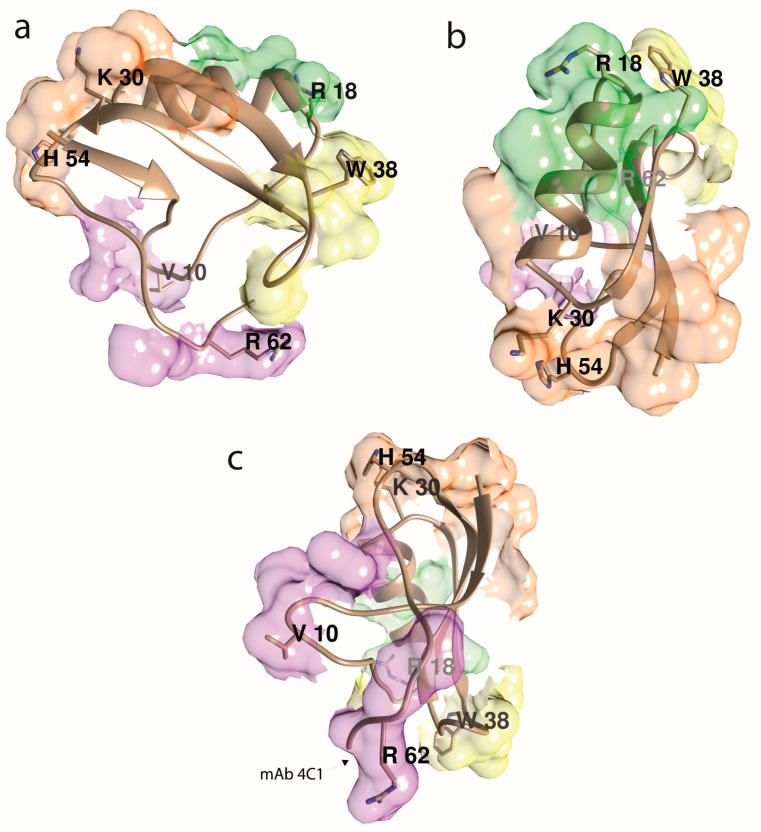
Aah II’s antigenicity, assuming that its four main antigenic epitopes are centered at the most protruding side chains, as indicated. The antigenic determinants (epitope area) are a mosaic of surface side chains that are adjacent in space but not necessarily within the toxin’s amino acid sequence. The illustration depicts all the immunological results compiled in review [82]. Molecular graphics were done with the UCSF Chimera package [91]. (**a**) β-sheets-oriented view. (**b**) α-helix-top-oriented view. (**c**) Cys12–Cys63 bridge-oriented view, opposite view as in (**b**). The mAb 4C1’s epitope (chiefly Arg62 and His_NH2_64) is noted [92].

In *Androctonus* venoms, neutralizing α-toxins by specific antisera completely inhibits their lethal activity. Unfortunately, the large antigenic polymorphism of these toxins, due to their wide structural polymorphism, complicates serotherapy. Scorpion α-toxins were first classified into four groups according to primary structure analogies; cross-neutralization and immuno-precipitation conducted using several specific antisera raised in rabbits against each highly purified toxin [76,77] (see also figure 3 in Reference [78]). These groups are the following:

(1) Group I, exemplified by Aah I, which also contains Aah III and Aah IV from *Androctonus australis hector*, Amm III from *Androctonus mauretanicus mauretanicus*, as well as Aam H1 and Aam H3 from *Androctonus amoreuxi*;
(2) Group II, with Aah II from *Androctonus australis hector* as protoype toxin, also contains Bot III from *Buthus occitanus tunetanus*, LqqV from *Leiurus quinquestriatus quinquestriatus*, AmmV and AmmVIII from *Androctonus mauretanicus mauretanicus*, and Aam H2 from *Androctonus amoreuxi*;
(3) Group III, which contains Bot I and Bot II from *Buthus occitanus tunetanus*, is the largest, because it contains almost all the α-like toxins from *Buthus*, and the α-toxins against insects, like Lqh αIT from *Leiurus quinquestriatus hebraeus*;
(4) Group IV, containing toxins similar to Lqq IV from *Leiurus quinquestriatus quinquestriatus*.

Sequence comparison revealed that less than 30% of similarity could be found between toxins belonging to different groups, whereas toxins may differ up to 50% within each group. This early classification was further substantiated using new techniques like enzyme-linked immunosorbent assay (ELISA) and liquid-phase radioimmunoassay (RIA) [79,80], and remained in accordance with the structural groups previously defined [77]. An antibody raised against a member of a structural–antigenic group is able to recognize and perfectly neutralize the toxins of the same group [79,80]. After four decades of research on *Androctonus* venoms, these affirmations are still without ambiguity, however, the structural polymorphism among the four recognized scorpion α-toxin groups remains a challenge for the preparation of efficient antisera and serotherapy improvement.

## 5. Epitope Mapping of *Androctonus* Toxins

### 5.1. First Attempts to Characterize Toxin Antigenic Sites

The number of antigenic sites on scorpion α-toxins has been determined by using *Androctonus australis* Hector toxins I and II (Aah I and Aah II) [80]. A minimum of four Fab fragments were found to bind simultaneously to each toxin. Taking advantage of the loss of a common antigenic site between Aah II and a highly homologous toxin from the same immunological group (Bot III, from *Buthus occitanus tunetanus*), an antibody population directed towards a single site of Aah II was purified and characterized. The site was found in the vicinity of the disulfide bridge Cys 12–Cys 63, which links the N-terminal to the C-terminal. Indeed, according to the Aah II 3D X-ray structure, this region is entirely exposed [72].

Next, the location of the main antigenic regions of Aah II was investigated. Using its available X-ray 3D structure, the relationship between structural elements and the location of its antigenic regions were analyzed. Reported antigenic sites in proteins correspond to segments that are particularly exposed and accessible. Accessibility and flexibility also show a strong correlation [81]. Thus, Aah II antigenicity was analyzed according to its segmental accessibility and flexibility. The hydrophilicity profiles of Aah II and 13 other α-toxins suggested that the antigenic sites were located in homologous regions. Some toxin hydrophobic conserved regions seemed to lack flexibility, mainly because of the disulfide bridge reticulation and a broad network of interchain hydrogen bonds. The antigenic Aah II regions were found to be located in exposed parts of the molecular surface, i.e., the reverse turns and the α-helix. They also correspond to segments of the polypeptide chain that are most accessible to a large spherical probe mimicking an antibody molecule. Looking to relationships between neighboring exposed residues within the protein led to the conclusion that, aside from classical sequence dependent epitopes (sequential epitopes), the main discontinuous antigenic determinants were found in scorpion toxins (conformational epitopes) [82]. Two main goals were then pursued at the same time: (i) the characterization of the toxin’s epitopes, and (ii) the preparation of the most performant antigen to improve the serum efficacy.

### 5.2. Use of Synthetic Peptides to Define Conformational Epitopes

Two peptides, which mimic two regions within Aah II that should be included in an antigenic site, were chemically synthesized, purified, and characterized. One of these peptides was situated around the Cys12–Cys63 bridge [80], and a second one at the 50–59 sequence in the C-terminal part [83]. Antibodies raised against the native Aah II individually recognized those peptides. Thus, these areas were thought to be involved in toxin antigenicity. These peptides were then linked to Sepharose in order to purify antibodies by affinity chromatography. Finally, selected antibodies were able to bind the ^125^I-labeled Aah II [84]. Using these data, it was thus established that the region around the disulfide bridge between Cys12 and Cys63 and the stretch of residues 50–59 most probably constitutes two antigenic sites, site 1 and site 2. Affinity chromatography isolated two antibody populations that were functionally independent. This result is not surprising, since the two regions carrying these two antigenic sites are not close to each other in 3D space and there is neither steric hindrance nor a cooperative effect between them.

The synthetic peptide 50–59, either free or bound to bovine serum albumin (BSA) was used to raise polyclonal antibodies, which can neutralize the Aah II effects in vivo. The antigenic specificities of the anti-peptide and antitoxin antibodies were compared with RIA by using ^125^I-Aah II in competition with chemically modified or homologous toxins. The anti-peptide antibodies showed a better affinity for the native Aah II than for the peptide 50–59 itself linked to BSA, and were completely unable to recognize the free peptide. This suggested that the restriction of the conformational freedom of the immunizing peptide was necessary in order to obtain high affinity anti-peptide antibodies for both the peptide and the toxin. In peptide 50–59, Lys58 was shown to be critical in maintaining Aah II toxicity but also 3D structural integrity [85]. According to these results, Lys58 appears to be an important immunogenic determinant [86].

The antigenicity of another peptide 19–28, corresponding to a rigid α-helix structure in the native Aah II 3D model, was tested in different solid phase RIA systems. Two subpopulations of anti-Aah II antibodies were purified by affinity chromatography, one on Sepharose–peptide 19–28, the other on Sepharose–Aah II. Native Aah II was recognized by the two subpopulations. However, these antibodies only poorly bound a denatured Aah II, suggesting that they identified the same antigenic surface as the antitoxin antibodies. This surface, corresponding to the α-helix, was thus conformation dependent, and corresponds to an Aah II conformational epitope [87]. The peptide 19–28 was also used as immunogen in its free form, or linked to BSA or to a macroporous polyacrylamide resin. Only the antibodies against the peptide bound to BSA or to the resin were able to recognize the native toxin. Finally, it was shown that the α-helix region remained accessible to these antibodies when ^125^I-Aah II was bound to Na_v_ channels in rat brain synaptosomes [88].

The sequences 19–29 and 28–39 of Aah II have also been chemically synthesized in order to study their four antigenic regions. These two peptides were chosen because of their high hydrophilicity values, and as such they were believed to play a role in toxin antigenicity. Two sub-populations from the total antibody pool raised against the native toxin were purified using affinity chromatography on each peptide. Still, they both bound to ^125^I-Aah II [89].

To summarize, using antibody populations raised against synthetic peptides and selected by immunoaffinity chromatography on these same peptides, four Aah II antigenic sites have been identified (Figure 3). The first one, surrounding the disulfide bridge 12–63, and a second one encompassing 50–59 residues are able to neutralize toxin toxicity. Fabs from the region around the disulfide bridge 12–63 inhibited ^125^I-Aah II binding to its Na_v_ channel binding site. On the contrary, when Aah II was already bound to its receptor, these two antigenic regions became inaccessible to their antibodies. However, two other antigenic regions, constituted by the α-helix (residues 23–32) and a β-turn (residues 32–35) remained accessible to their respective antibodies when the toxin was bound to its receptor [90].

### 5.3. The Pepscan Method

New software was developed for the design of peptides prepared by the manual Spot synthesis method (Pepscan) [93]. In order to map the antigenic reactivity pattern of Aah II, a set of 58 overlapping rod-bound peptides was used. Six sequence-dependent antigenic regions were identified (sequences: 1–8, 4–12, 27–35, 39–45, 52–58, and 55–61). These regions correspond to either the β-turn or extended parts of the molecule, as already mapped by other methods mentioned above. An important discrepancy was, however, that the Aah II N-terminus was found to be strongly reactive with rabbit anti-Aah II antibodies. Thus, a synthetic peptide mimicking the Aah II sequence 1–8 was used to raise antibodies, which bound the toxin with high efficiency. Peptides corresponding to an Ala-scanning of the sequence 1–7 and peptides designed according to the N-terminal of the other *Androctonus* toxins were analyzed with the anti-Aah II and anti-peptide (1–8) antibodies. The crucial residues in the Aah II N-terminal recognition were: Lys 2, Asp 3, and Gly 4 [94].

### 5.4. Use of Monoclonal Antibodies to Characterize Androctonus Toxins Epitopes

Monoclonal antibodies (mAbs) have been helpful for studying the antigenic properties of *Androctonus* toxins for epitope characterization in view of antivenoms preparation. To obtain mAbs, the animal spleen is isolated after a set of booster injections. Next, the lymphocytes are immortalized by cell fusion and then screened for high affinity antibody binding. The hybridomas producing the chosen antibodies can then be expanded, and the mRNA encoding the heavy and light chains isolated and mutated to further enhance the affinity of the antibody. Several mAbs were generated in order to neutralize Aah II. These studies will be discussed in Section 6.8.

## 6. Research Studies to Improve Antibody Preparation

### 6.1. Detoxification by Chemical Modification

The first attempts were made using four different Mexican *Centruroides* species. Four different immunogens (i.e., crude venom, telson extract, toxic fraction obtained from the telson extract by gel filtration, and the same toxic fraction modified using acetic anhydride) were injected in rabbits in order to prepare antisera [95]. After acetylation, the obtained proteins were nontoxic. Even though twice as much immunogen was injected in the animals, the neutralizing capacities in mice (LD_50_/ml of serum) of the antiserum obtained with the acetylated toxic fractions were slightly weaker than those obtained with the non-acetylated fraction. We anticipated a poor recognition of the native toxins by the antibodies, following conformational perturbations of the antigenic sites after chemical modification. However, an atoxic form preparation offers clear advantages. 

### 6.2. Venom Trapped in Liposomes

Raising a humoral immune response in mice in order to protect them from the lethal effects of scorpion toxins was evaluated by using a venom toxic fraction trapped in nontoxic sphingomyelin–cholesterol liposomes. Mice with the highest levels of circulating antitoxin antibodies finally survived the challenge by twice the LD_50_ of the toxic fraction, which killed all control non-immune mice. This in vivo protection was, however, found to be limited both in its duration and its effectiveness against higher amounts of toxin [96]. 

### 6.3. Use of Synthetic Peptides or Synthetic Aah II to Generate Neutralizing Antibodies

The possibility of eliciting toxin reactive antibodies by immunization with short peptides was assessed. The sequence 50 to 59 of Aah II was chosen according to the aforementioned results (see Section 5.2). The synthetic peptide was used in different forms (free, linearly polymerized, coupled with KLH, coupled with a low-molecular weight B-lymphocyte activator) in order to immunize different groups of C57BL/6 mice. ELISA was used to characterize serum reactivity. Both the peptide (mean titer over 1:52,600) and the toxin KLH-coupled peptide (mean titer 1:800) gave a strong reactivity. Unfortunately, the immune sera produced were unable to neutralize the lethal effects of the toxin. Further studies using liquid-phase RIA showed that the native toxin was not recognized, indicating that the neutralizing properties depend on the Aah II conformational epitopes [97].

### 6.4. Chemically Synthesized Aah II Variant Devoid of Cysteines Bridges

At first, glutaraldehyde polymerization of the *Androctonus* toxins was used to obtain antigen aggregates with decreased toxicity [98]. Then, a chemically synthetic (Abu)8–Aah II, with each half-cystine being replaced by the isosteric residue α-aminobutyric acid and thus devoid of disulfide bridges, was used as antigen in mice and rabbits. This construct was totally nontoxic in mice even if large amounts, equivalent to 1000 times the LD_50_ of the native toxin, were injected. The monomeric or the glutaraldehyde-polymerized Aah II was able to protect the immunized animal against several mortal doses of the unmodified Aah II. The protection was better when the polymerized synthetic peptide was used. The antibody titer decreased with time; however, the immuno-protection remained high six months after immunization [98].

### 6.5. Use of Native Anatoxin to Generate Antiserum

A natural anatoxin from *Androctonus mauretanicus mauretanicus*, AmmVIII, which shares 87% identity with Aah II, was used to generate antisera in rabbits. The elicited antisera were able to protect mice against Aah II. The anti-Amm VIII sera prevented the association of ^125^I-Aah II with Na_v_ channels in rat brain synaptosomes, and also removed ^125^I-Aah II already bound to its binding site [99]. As AmmVIII antibodies were supposed to recognize discontinuous epitopes, 24 peptides mimicking discontinuous regions of AmmVIII were designed in silico and prepared by Spot synthesis. Anti-Amm VIII antibodies recognized seven of these discontinuous–continuous peptides. The 3D location of the segments constituting the antigenically reactive discontinuous–continuous peptides pointed out three Amm VIII regions quite similar to the Aah II 3D structure [100]. Region A was in the α-helix and the three-stranded β-sheet of the toxin. Region B comprised segments from a β2–β3 loop. Region A and B antigenicity in the Aah II toxin had already been experimentally assessed. The Amm VIII region C was made of different segments from the conserved hydrophobic surface described in the α-toxins. It included positions 10 and 58 that were found in the discontinuous epitope of the 4C1 anti-Aah II monoclonal antibody (mAb), as well as three Tyr residues belonging each to a separate strand of the β-sheet.

### 6.6. Androctonus Toxins as Fusion Proteins Expressed in Escherichia coli

Recombinant toxins from Aah I, Aah II, and Aah III, as well as a Aah I–Aah II tandem construct, all produced as fusion proteins with maltose binding protein (MBP) in *Escherichia coli*, were used as immunogens in rabbits and mice in order to obtain protective antisera against the most lethal α-type toxins in the Aah venom. In vivo, the fusion proteins induced a long-term protection in mice against the lethal effects of the native toxins [101]. However, no effective vaccine was proposed in these studies [102].

### 6.7. Phage Display

More recently, the well-established phage display method enabled extensive toxin epitope studies [103]. By screening peptide libraries (‘bio-panning’) with mAbs or with serum, peptides mimicking antigenic and immunogenic specificity of their epitopes were characterized. Selected epitope mimics constitute functional versions of native epitopes, and are efficient means of elucidating the epitope structure and location. Induction of immune responses against the antigens they represent can then be made. This phage display technique can also help identify epitope elements that are crucial for binding and are represented by a small number of amino acid residues mimicking epitopes, termed ‘minimal epitope.’ Thus, the phage display technique is used as a viable alternative method to in vivo immunization for raising antibodies.

### 6.8. Monoclonal Antibodies

Several mAbs were generated in order to neutralize Aah II. One of them (the mAb 4Cl) was able to neutralize toxin binding to its receptor in a competitive way (Kd = 0.8 nM), due to the epitope overlapping or being close to the receptor-binding region of the toxin. It recognized several residues of the toxin clustered in the C-terminal and showed a robust neutralizing effect in mice (1 mg of the mAb neutralized 30 LD_50_ doses). Its neutralizing activity could be related to the positive charge of the Lys58 side chain. The mAb binding abolishes the toxicity, either by preventing the interaction with the membrane or by the acceleration of the dissociation of the pre-bound toxin from its receptor [104]. The epitope specificities of mAb 4C1 and of a second mAb, mAb 3C5 were then characterized using 58 overlapping linear synthetic heptapeptides, which covered the whole sequence of Aah II. None were able to recognize any of the heptapeptides. Therefore, conformation-dependent epitopes at the surface of the toxin were thought to be recognized by both mAbs. Further experiments were designed to see whether or not the two mAbs were capable of binding simultaneously to the toxin. It was found that the epitopes recognized by mAb 4C1 and mAb 3C5 were close together at the Aah II surface, thus preventing the simultaneous binding of both mAbs to a single toxin molecule [105]. The synthetic nontoxic analog (Abu)8–Aah II was also used to obtain mAbs, which recognized and neutralized the native toxin. Sets of peptides spanning the entire Aah II sequence allowed the identification of different sections of the native Aah II sequence, suggesting that several regions of the (Abu)8–Aah II sequence mimic the native Aah II epitopes and can produce mAbs directed against the toxin [106].

However, no mAb production was obtained in mice injected with the other major lethal toxin of the Aah venom, Aah I, in a native or a chemical inactive (Abu)8–Aah I form. A new strategy in which Aah I was coupled to a carrier protein (BSA, KLH, or the nontoxic analog of Aah II) led to a panel of high-affinity mAbs. The mAb 9C2 was particularly interesting, since it was also reactive with other toxins classified into the same immunological group as Aah I and Aah III. It was capable of neutralizing the toxic effect of the parent toxin as well as the venom. It showed a 150 pM affinity for the toxin, and was used to set up a two-site ELISA for the quantification of Aah I in the biological fluids of envenomed animals. The detection limit of the assay was 75 pg/ml. Thus, the anti-Aah I mAb 9C2, associated with anti-Aah II mAb 4C1, constituted a new valuable tool in serotherapy [107,108].

In spite of the clear neutralizing potential of these mAbs, enthusiasm about their therapeutic value was diminished by their murine origin. Therefore, single-chain derivatives (scFv) of the mAbs 4C1 and 9C2 variable regions have been engineered and expressed in *Escherichia coli*. Polymerase chain reaction (PCR)-mediated cloning allowed the isolation of the cDNAs encoding the variable regions of mAb 4C1. Fv fragments are the minimal structure to retain the binding characteristics of an antibody, and can be produced in heterologous system. The recombinant variable domains VH and VL were genetically linked in a single chain (scFv) construct. A small peptide connects the C-terminal of VH to the N-terminal of VL and stabilizes the association. The recombinant protein showed pharmacological and biological activities similar to those of the initial antibody both in vitro and in vivo [109]. Due to their small size, these molecules can diffuse into extra vascular compartments and are more effective than mAb. However, they are rapidly degraded, and their monovalent binding decreases their commercial viability. To circumvent these restrictions, a product from a non-covalent association of two scFv (diabody) was designed [110]. A recombinant functional diabody, designed from mAb 9C2, which recognized Aah I, was produced in the periplasm of *Escherichia coli*. It displayed 80 pM affinity for Aah I, showed a high thermal stability in serum and a protective activity in challenged mice. However, the diabody cannot be produced in non-bacterial systems in amounts required for practical use. However, a unique bispecific tandem-scFv able to neutralize both Aah I and Aah II was produced in bacteria. It was able to neutralize the binding of the two toxins to their receptor and, in vivo, protected mice against experimental envenomation [111]. Also, the diabody (anti-Aah I) Db9C2 and the diabody (anti-Aah II) Db4C1 were mixed in a way that the molar ratio matched the characteristics of toxins and polymorphism in the venom. In vivo, under conditions simulating scorpion envenomation, the intraperitoneal injection of 30 μg of the diabody mixture protected almost all the mice exposed to 3 LD_50_ of venom injected by subcutaneous route. The presence of both diabodies was necessary for the animals to survive [112].

Finally, to better define the epitopes and pharmacological sites of Aah II, crystallographic analysis was performed using an antigen-binding fragment (Fab), the anti-Aah II mAb 4C1 having high-affinity and selectivity for Aah II [104]. The crystal structure of the Fab–Aah II complex was solved at 2.3 Å resolution [92]. The binding of Aah II onto the Fab4C1 variable region was surprisingly complementary in shape, chemistry, and electrostatic potential of the Fab paratope surface, negatively charged, and the toxin epitope surface, positively charged. Bound Aah II was oriented with the C-terminal pentapeptide region, and non-continuous residues from the long β1–α1 segment and the β2–β3 turn, deeply buried in the binding pocket (25% of its molecular surface). The residues Arg56 to His64 contributed to 68% of the binding surface (Figure 3c). The overall conformation of bound Aah II was unchanged compared to unbound Aah II, except the C-terminal Arg62 to His64, where a 75° rotation reduced the flexibility of the Cys12–Cys63 bridge, and thus drastically modified the backbone direction.

From the anti-Aah I mAb 9C2, a crystallization step of a high affinity Aah I–Fab complex was also performed [92]. However, the resulting 1.6 Å resolution structure revealed only a Fab molecule devoid of bound Aah I, denoting the expulsion of the bound antigen upon crystal formation. Comparative analysis and complementary data from a flexible docking study proposed the occurrence of distinctive trapping orientations for the two toxins, relative to their respective Fab.

### 6.9. Camelid

Immunization of dromedaries (*Camelus dromedarius*) with the weakly antigenic *Androctonus australis* toxins was deemed a success [113]. Camel immune sera neutralized both the venom toxic fraction and Aah II toxin. A significant proportion of polyclonal heavy chain antibodies recognized the toxins, in particular Aah II, and neutralized their toxicity in mice [113].

Later, an alternative to classical antivenoms was proposed by using nanobodies (single-domain antigen-binding fragments derived from dromedary heavy-chain antibodies). A VHH (variable domain of the heavy chain of a HCAb (heavy-chain antibody)) antibody against the toxin Aah I was obtained from an immune library and phage display technology. Diverse formats from this VHH, which neutralized the toxic Aah I effect, were constructed [62]. These nanobodies were stable, and distributed rapidly and easily into envenomed animals. All these constructs possessed a scorpion toxin neutralization capacity in mice that far exceeded the previous attempts achieved with scFv-based materials. A controversial point is that this experiment was conducted by administering the antibodies by i.c.v. injection, making it difficult to correlate their affinity with their protective capacity, since neuronal Na_v_ channels are not necessarily present in the organs targeted by the toxins in the peripheric system during a real case of a scorpion sting. However, we have to notice the presence of neuronal sodium channels in the periphery (for example, Nav1.1 [114]).

Additionally, multiple other nanobodies of sub-nanomolar affinity against Aah II were isolated from a dromedary. One of them, referred to as NbAahII10, showed a neutralizing capacity in mice of about 37,000 LD_50_ of toxin/mg of nanobody [115]. Furthermore, a mutant NbAahII10 Cys/Ser, where Cys38 was replaced by Ser in order to avoid dimerization upon prolonged storage, was subsequently humanized. It maintained a high affinity for Aah II, and its neutralizing capacity was preserved without conceding much expression yield and stability [116]. A structural–computational approach was recently conducted to elucidate Nb–Aah II interactions [117] using an Nb multiple sequence alignment, followed by modeling and molecular docking with Rosetta-Antibody-RAbD [118] and ZDOCK [119]. Finally, a couple of NbAahII10–Aah II residue interactions (Gln38–Asn44 and Arg62–His64, respectively) emerged from the study, and were found to be mainly involved in the toxic Aah II binding site, in accordance with the crystallographic data as previously described above [92].

A bispecific NbF12–10 was designed against Aah I/Aah II toxins. A subsequent intravenous injection of 85 μg of NbF12–10 protected all mice subcutaneously injected with a lethal dose of Aah venom. Furthermore, when mice with severe signs of envenoming were treated, the NbF12–10 remained fully protective, whereas the untreated mice died [120]. The pharmacodynamics of NbF12–10 were investigated in rats by in vivo echocardiography. NbF12–10 was able to inhibit the hemodynamic disorders provoked by a lethal dose of venom. It restored heart rate and blood pressure values, and prevented lung and heart lesions of treated mice after envenoming. The authors suggested that the nanobody based therapeutic could replace classic Fab'(2) based products as immunotherapeutic in scorpion envenoming [121]. Altogether, all these efforts led to valuable insights in the design and development of the future antivenom.

## 7. Advanced Scorpion Envenomation Therapies

Despite progress in understanding the pathophysiology of scorpion envenomation and its treatment, challenges still remain [122]. As such, the development of effective, cheap and safe treatments is greatly needed. Understanding the cellular and molecular events involved offers promising future therapeutic and diagnostic opportunities. Such possible therapeutic targets include adhesion molecules, matrix metalloproteases (MMPs), and inflammatory cytokines and their receptors. Given the potential involvement of oxidative stress in the pathogenesis of envenoming, there are reasons to believe that antioxidant compounds might also be beneficial.

Nowadays, antivenom obtained from hyper-immune horses is the only treatment used for envenomed humans. However, current limitations of serotherapy require an efficient alternative with a high safety margin, target affinity, and more promising venom neutralizing capability [123].

Neurotoxins are major lethal constituents of scorpion venoms, and are found in relatively low abundance in the venom (i.e., about 3–10 % in weight for an electrically obtained crude venom). Moreover, because of their small size, they are frequently not able to elicit a strong immunogenic reaction. As such, they may not always induce the production of high-quality and sufficient quantity of antibody molecules. Consequently, a balance between the injected doses, the toxicity towards the animal and high-quality antibody production has to be obtained, often empirically.

In spite of the cost and time needed, isolation of the main toxic venom components to develop antisera has advantages, compared to using the crude venom as an antigen to raise therapeutic antibodies. Purified neurotoxins constitute a remarkable tool for the preparation of highly protective antivenom. In this case, isolated antibodies are specific to the scorpion toxin family injected. Such antibodies are useful, for example, in the detection and isolation of scorpion toxin polypeptides present in biological samples.

A complicating factor in antiserum design is reports of controversial results stemming from the use of different antidote preparations in trials and clinical practice against local scorpion species. As a result, there is an absence of consensus among researchers. African and Brazilian authors note that the availability of potent and reliable antivenom sera in these regions is limited [102].

The improvement of the serotherapy efficiency could be based on the immunization protocol, including type of antigen (venom vs. toxoid), adjuvant type, immunization dose, frequency and intervals of immunization, and animal choice (horse vs. camelid or chicken). The choice of animal producer is an important factor in the generation of effective antibodies. The use of mammals seems to be ineffective due to the secondary reactions, including anaphylactic shock. Other strategies using animals phylogenetically distant from mammals could avoid these reactions [124]. A camel immunized with the toxic major fraction from Aah venom led to a high titer in conventional IgG and HCab (heavy-chain-antibody), lacking light chain and CH1 domain. Obtained serum was able to successfully neutralize venom toxicity [113]. Fragments of recombinant VHH antibodies obtained from HCab antibodies expressed in recombinant bacteria were characterized by very high stability, low immunogenicity, and high production [120,121,125]. However, some properties of VHH needed improving in order to be fully implemented. Affinity, specificity, and size properties were functional limitations in pharmacogenetics of VHH. Additionally, the therapeutic efficiency of VHH was also limited by the short serum half-life [126].

Another alternative that has proved its usefulness in experimental therapy is the use of IgY obtained from egg yolks [127,128,129]. Compared to mammals, chickens are economically attractive in terms of polyclonal antibody production, since antibody levels are higher (5–6 times more than a in a rabbit over a 2-week period) and production lasts a long time, since a laying hen can be productive throughout the laying period and beyond [130]. This economic benefit is reinforced by: (i) the lower cost of feeding and housing chickens, compared to other animals such as horses or sheep, and (ii) the ethical production of IgY from the egg yolk [129,131]. Recently, it has been reported that IgY antibodies effectively neutralize the Aah venom lethality, and could prevent its pathological effects [132].

It is worth noting that a prophylactic treatment could be used to prevent the induced pathophysiological effects and the lethality of scorpion envenomation [133,134,135]. The development of a vaccine-like product could enhance the immune responses to a specific antigen without deleterious side effects caused by adjuvants [136]. It was reported that the Aah venom and its toxic fraction FtoxG50 vaccine nano-formulations present a high immunogenicity, an important immune-protective effect, and a low reactogenicity [137,138].

Finally, all these academic studies showed that, from a practical point of view, laboratories producing antivenoms should use at least partially purified venom fractions, or a mixture of pure antigens, to obtain antivenoms able to neutralize neurotoxins belonging to the different structural and immunological groups. Serotherapy as a specific treatment is usually recommended after scorpion stings in most of the regions at risk. However, to be of better effectiveness, this therapy needs to be improved, optimized, and standardized considering the limitations (delay, antibody format, soluble or freeze-dried, dose, route of injection, etc.), and also the scientific and technological advances.
